# Effect of Genital Sampling Site on the Detection and Quantification of* Ureaplasma *Species with Quantitative Polymerase Chain Reaction during Pregnancy

**DOI:** 10.1155/2017/6725168

**Published:** 2017-02-05

**Authors:** Gilles Faron, Ellen Vancutsem, Anne Naessens, Ronald Buyl, Leonardo Gucciardo, Walter Foulon

**Affiliations:** ^1^Department of Obstetrics and Prenatal Medicine, Universitair Ziekenhuis Brussel, 101 Laarbeeklaan, Jette, 1090 Brussels, Belgium; ^2^Department of Microbiology, UZ Brussel, 101 Laarbeeklaan, Jette, 1090 Brussels, Belgium; ^3^Department of Biostatistics and Medical Informatics, Faculty of Medicine and Pharmacy, Vrije Universiteit Brussel, 101 Laarbeeklaan, Jette, 1090 Brussels, Belgium

## Abstract

*Objective*. This study aimed to compare the qualitative and quantitative reproducibility of quantitative PCR (qPCR) for* Ureaplasma *species* (Ureaplasma *spp.) throughout pregnancy and according to the genital sampling site.* Study Design*. Between 5 and 14 weeks of gestation (T1), vaginal, fornix, and two cervical samples were taken. Sampling was repeated during the 2nd (T2) and 3rd (T3) trimester in randomly selected T1 positive and negative women. Qualitative and quantitative reproducibility were evaluated using, respectively, Cohen's kappa (*κ*) and interclass correlation coefficients (ICC) and repeated measures ANOVA on the log-transformed mean number of DNA copies for each sampling site.* Results*. During T1, 51/127 women were positive for* U. parvum* and 8 for* U. urealyticum *(4 patients for both). Sampling was repeated for 44/55 women at T2 and/or T3; 43 (97.7%) remained positive at the three timepoints. *κ* ranged between 0.83 and 0.95 and the ICC for cervical samples was 0.86.* Conclusions*. Colonization by* Ureaplasma *spp. seems to be very constant during pregnancy and vaginal samples have the highest detection rate.

## 1. Introduction

Preterm delivery, defined as birth before 37 weeks, is responsible for 75% of perinatal mortality and for more than 50% of the long-term morbidity among survivors [1]. Spontaneous premature labor or premature preterm rupture of the membranes is involved in 60–65% of premature births [2]. These outcomes could be improved with development of specific care and with more accurate identification of pregnant women with obviously increased risks of premature delivery among the heterogeneous population of more or less symptomatic patients.

Evaluation of the vaginal flora during pregnancy has been pointed out as a potentially interesting screening tool in this context [3, 4]. Although considered to be commensal in the female genital tract, the presence of* Ureaplasma* species* (Ureaplasma *spp.) during pregnancy is independently associated with an increased likelihood of clinical chorioamnionitis, preterm premature rupture of membranes (PPROM), and preterm delivery [5, 6].* Ureaplasma *spp. induce inflammatory response in chorion and amnion cells [7] and contribute to collagen fragmentation which weakens amniotic membranes [8].

Two methods have been described to screen for* Ureaplasma *spp.: culture is the gold standard, but this method gives no information about the different species (*Ureaplasma urealyticum (U. urealyticum)* or* Ureaplasma parvum (U. parvum)*) and gives few indications of their quantity. The second method, quantitative polymerase chain reaction (qPCR), has the huge advantage over culture of being able to differentiate* U. urealyticum* and* U. parvum*. Studies on the pathogenicity of* Ureaplasma *spp. in pregnancy have addressed the possibility that there may be a difference in pathogenic potential among species [9]. Moreover, bacterial load could also be a factor which enhances the invasive potential of the microorganism [5]. Culture based studies have reported that women harbor different bacterial populations in the cervix than in the vagina and that the vaginal flora was a dynamic ecosystem [10]. One study has investigated the constancy of the burden of* Ureaplasma *spp. in genital secretions [11] throughout pregnancy, but none has reported the distribution of* U. urealyticum* and* U. parvum* during pregnancy. The influence of the sampling location on the detection and quantification of* Ureaplasma *spp. has also not been studied previously. Therefore, the aim of this study was to compare the qualitative and quantitative reproducibility of a qPCR technique used to identify the presence of both* Ureaplasma *spp. according to the sampling site, within the same woman, throughout pregnancy. As a secondary outcome, we studied the epidemiology of* Ureaplasma* species (*parvum* or* urealyticum*) during pregnancy and the evolution of the colonization of the women, using three timepoints.

## 2. Material and Methods

This was a prospective study conducted in the antenatal outpatient clinic of the Universitair Ziekenhuis Brussel (UZ Brussel) from February 2011 to January 2015. Healthy women with uncomplicated pregnancies were invited to participate. During the first prenatal visit that was scheduled before 14 weeks of gestation, we gave oral information about the study. Written informed consent was obtained from those agreeing to participate. This study was approved by the hospital's ethics committee. Gestational age was ascertained from the last menstruation date and a first trimester ultrasound examination. The participants were tested between 5 and 14 weeks of gestation. The exclusion criteria included patients under 18 years old, clinical vaginal infection, or documented recent intake of antibiotics (whatever the reason).

After exposing the cervix with a sterile speculum, four samples were taken for PCR quantification of* U. parvum* and* U. urealyticum*, in the following order: vaginal, fornix, and two from the (exo)cervix (cx1 and cx2). Sampling was performed gently to avoid (cervical) bleeding. The samples were transported in the “Universal Transport Medium System” (UTM) (Copan Italia, Italy, containing 3 ml liquid medium) and processed using a qPCR method that was described in a previous publication [12].

We recruited women during the first trimester and they were considered positive if any of the four genital tract samples tested was reactive in qPCR. We intended to repeat the same sampling during the second trimester (between 18 and 27 completed weeks, T2) and during the third trimester (between 28 and 39 weeks, T3) for these positive women. The lack of available publications about this specific research topic made the required sample size impossible to calculate accurately.

For those who were negative at the first sampling we decided to test some of them again, at random, during the second and the third trimester to confirm that the absence of* Ureaplasma *spp. was maintained throughout the pregnancy.

Qualitative reproducibility was assessed by comparing the proportions of qPCR positive tests for each sampling site with a Fisher's exact test, both at the first trimester and in a second analysis for all samples (T1, T2, and T3). Cohen's kappa coefficient (*κ*) was used to assess the degree of interrater agreement between the different sampling sites. The agreement between two observations is generally considered excellent when *κ* > 0.75 [13].

The quantitative reproducibility of the test was assessed at two levels among the patients who were found positive:

1. We calculated the mean number of DNA copies for each sampling site. In order to normalize our results and minimize the outlier effect we used (base 10) logarithmically transformed data. The computed mean log of the two cervical samples was used to compare with those from other sampling sites, except if one of them was negative; in that case we took into account only the value of the positive result. A repeated measures ANOVA, with pairwise comparisons (Bonferroni corrections), was used to analyze the quantitative differences among locations as well as potential differences in carriage among T1, T2, and T3.
2. To test the degree of quantitative agreement between two samples taken simultaneously from the same location, we considered the two repeated samples taken from the cervix during all trimesters (when both of them were positive) and computed the interclass correlation coefficient (ICC) and its 95% confidence interval (95% CI).

All calculations were carried out using IBM SPSS version 22.0. Baseline data were summarized for each group and compared (comparison of two proportions or two means, with 95% CI). Unpaired *t*-tests, the two-sided Fisher's exact test, and 95% CI were used when appropriate. A *P* value < 0.05 was considered to be statistically significant.

## 3. Results

**Figure 1:**
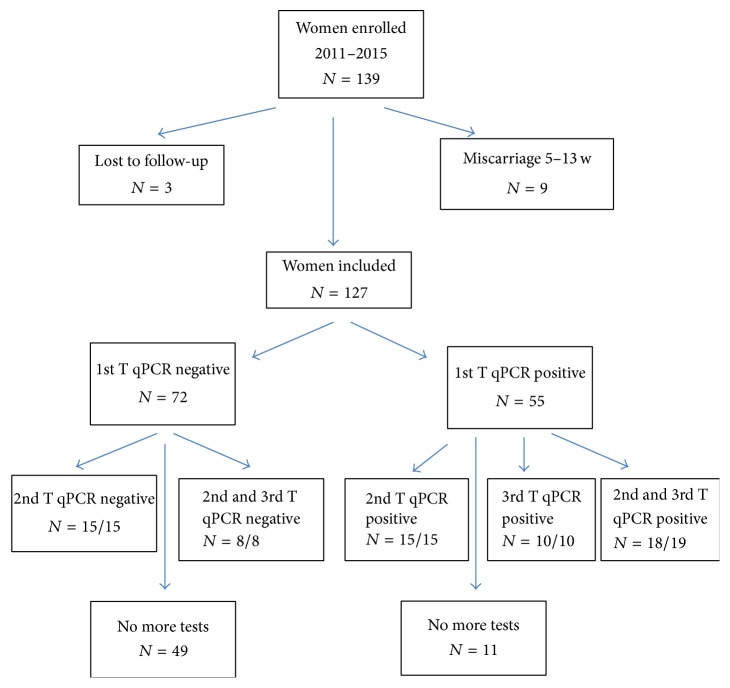
Enrolments of participants.

We recruited 139 women, who were tested between 5 and 14 weeks of pregnancy. Three were lost to follow-up and were excluded from our analysis (Figure 1). Nine women had a miscarriage between 6 and 13 weeks: among them, six were positive for* U. parvum* for all sites, including one patient with associated positivity for* U. urealyticum* in the fornix and the vagina. The three other women had negative results for all sampling sites.

Of the remaining 127 women, 72 (56.7%) had negative qPCR results for all sites and 55 (43.3%) were positive for at least one location during the first trimester. Follow-up samples were taken from the three different locations for 23 of the 72 negative women; 15 were tested in the second trimester and 8 other patients were sampled in both the second and the third trimester. All these women remained negative at all sampling timepoints.

Among the 55 women with +qPCR, 51 (92.7%) were positive for* U. parvum* and 8 (14.5%) were positive for* U. urealyticum*; 4 (7.2%) of these women were positive for both species.

Among the 51 women positive for* U. parvum*, 43 were positive at all locations, two were positive only in the vagina, four were positive in the vagina and the fornix (but negative for both cervical samples), and two had discordant results in the cervix. There was a statistically significant lower mean bacterial load (5.08 log copies/mL (95% CI: 4.58–5.59)) in the group of eight women not positive for all locations, in comparison with the mean bacterial load of the 43 women for whom all four samples were positive (5.95 log copies/mL (95% CI: 5.70–6.20)) (*P* = 0.008).

Among the eight women positive for* U. urealyticum*, one was positive only in the vagina and three had discordant results in the cervix; the other four were positive in all locations. The same trend for a lower* U. urealyticum* load in the vagina was noticed in the four women with discordant results (as observed for* U. parvum*) but the difference did not reach the statistical significance probably due to the small groups (4.58 log copies/mL (3.87–5.30) versus 5.43 (4.08–6.79)), *P* = 0.32.

Among the 55 women who showed +qPCR in the first trimester, 44 were retested later in pregnancy, either in the second trimester (15 women) or during the third trimester (10 women) or during both second and third trimesters (19 women). Among all these positive women, only one woman with an uneventful pregnancy showed discordant qualitative tests results during her pregnancy. This patient had a +qPCR at T1 and T2 (with mean log-transformed values that were not statistically different from those of the other patients) but negative qPCR at T3, with no history of antibiotic treatment in the meantime.

The distribution of the positive samples according to the sample location and the sampling timepoint during pregnancy is detailed in Table 1.

The qualitative analysis of the qPCR results with comparison between locations using Cohen's kappa coefficients (*κ*) revealed high agreement, with *κ* values between 0.83 and 0.95 (Table 2). The results for vaginal and fornix samples showed the highest agreement.

Eighty-four positive repetitive cervical samplings (T1: *n* = 42; T2: *n* = 25; T3: *n* = 17) were used to test the quantitative agreement between the two cervical sampling locations. The ICC value was 0.86 (95% CI: 0.73–0.92) which should be considered to show good agreement, according to the literature [13].

**Figure 2:**
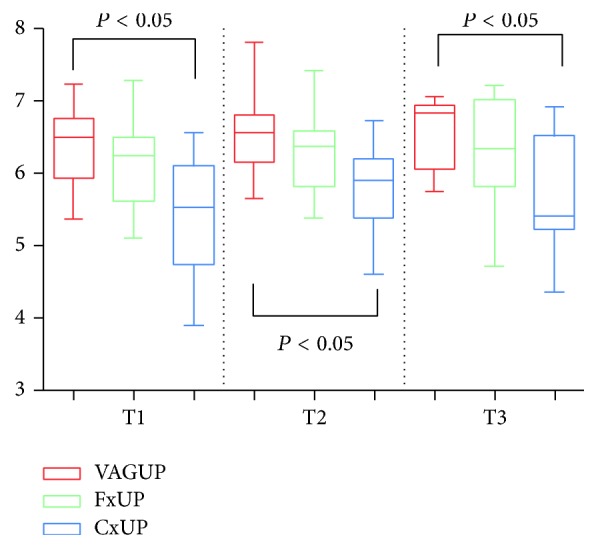
Mean log values (with 95% CI) of* U. parvum* (UP) during pregnancy for the three sample sites. VAG UP: vaginal* Ureaplasma parvum*. Fx UP: fornix* Ureaplasma parvum*. Cx UP: cervical* Ureaplasma parvum*. T1: first trimester. T2: second trimester. T3: third trimester.

The evolution of the quantitative qPCR results during pregnancy is summarized in Figure 2 using mean log-transformed values for each sampling site for* U. parvum* at T1, T2, and T3. This analysis was not done for* U. urealyticum* because there were too few positive results.

For each trimester, the mean number of DNA copies for* U. parvum* was highest for vaginal sampling; the lowest numbers were observed for cervical sampling. The fornix burden showed intermediate results. This statistically significant trend was still observed after exclusion of women with negative qPCR results in the cervix, confirming the lower values for cervical microbial load. A trend was observed, showing increasing log-transformed values for* U. parvum* with time during pregnancy. This was seen for all sample locations but did not reach statistical significance.

## 4. Discussion

Colonization with* Ureaplasma *spp. was found in 43.3% of the women. Colonization with* U. parvum* was far more frequent than with* U. urealyticum* (40.2% versus 6.3%) and this correlates very well with previous studies [12, 14, 15]. Using a different methodology, Knox and Timms concluded that* Ureaplasma *spp. (formerly named* Ureaplasma urealyticum* subdivided into different serovars) were often persistent colonizers of the lower genital tract from mid-pregnancy until the third trimester of pregnancy [11]. Our study, using a more recently developed qPCR technique, confirms this and gives more information about the constancy and the density of* Ureaplasma *spp. in genital secretions from an early gestational age to the end of pregnancy and at different sampling sites.

In our study, positivity as well as negativity for* Ureaplasma *spp. carriage was almost constant during pregnancy, without any intervention. All negative women remained negative throughout the pregnancy, and 98% of the women with a +qPCR result in the first trimester were confirmed with positive samples at T2 and/or T3. These findings are essential for clinicians, because it implies that, in order to study the pathogenicity of* Ureaplasma *spp. during pregnancy, there is no need to use a standardized screening timepoint at a certain gestational age. Therefore one can presume that patients who are positive at a certain time during pregnancy were or will be positive throughout the current pregnancy in the absence of antibiotic intake. Our study was underpowered to be able to analyze outcomes like preterm birth or neonatal complications but to avoid bias we have only tested low-risk pregnant women.

The real-time qPCR for* Ureaplasma *spp. demonstrated excellent qualitative agreement whatever the site of sampling (*κ* values between 0.81 and 0.95). Nevertheless the probability of a positive qPCR result is significantly greater for a vaginal sample when compared with other sampling sites. This particular trend was observed during all three trimesters for* U. parvum* and at least during the first trimester for* U. urealyticum*. A group of eight women (14.5%) had a positive vaginal qPCR result but negative cervical qPCR. Analyzing the bacterial load in these discordant samples, we found a lower mean vaginal bacterial load when compared with women with positivity at all locations. Remarkably, among the 4/8 women retested later (T2 and/or T3), 3/4 showed the same profile: +qPCR in the vagina and fornix, but still negative in the cervix. We can conclude that the vaginal burden has an impact on the colonization of the cervix: the higher the vaginal bacterial load, the higher the risk of positive cervical colonization. The vaginal colonization should be considered as a “reservoir” allowing secondary colonization of the cervix and further ascending infection in function of different local conditions like change of pH during pregnancy or decline in maternal immunity.

Quantitative analysis of* Ureaplasma *spp. colonization performed at T1, T2, and T3 did not identify significant patterns of variation: the observed trend for augmentation during pregnancy remained lower than 1 log for* U. parvum*. The small number of positive cases for* U. urealyticum* prevented any definitive conclusions for this species.

Overall, the colonization by* U. parvum* was quantitatively greater in the vagina than in cervical samples (Figure 2). These differences reached 1 log (or were very close to 1 log) in almost all the cases, and this may explain why in some cases the qPCR was positive in the vagina and negative in the cervix. These challenging findings may have clinical implications, especially if we wish to optimize the identification of pregnant women with a high risk of late miscarriage or preterm labor using this qPCR technique.

Therefore, several questions deserve to be asked: Do we need to sample the vagina, the cervix, or both? How should we interpret a positive vaginal sample associated with a negative cervical one? The fact that the cervix is not colonized despite a positive vaginal sample could reflect a better immune response with a decreased associated risk of adverse outcomes due to* Ureaplasma* colonization. This is an interesting hypothesis to be tested using a population of pregnant women with negative cervical qPCR combined with a positive vaginal sample. Our study was not powered to answer this clinical question, but we intend to develop a new experimental design based on these findings. Depending on these future results, two screening options could be assessed: a vaginal screening method (easier, no speculum needed) recruiting more positive women and a cervical screening procedure, technically more difficult to perform but recruiting patients at potentially higher risk.

We believe that screening for* Ureaplasma* during pregnancy combined with species identification and quantification should be a new direction of investigation to better understand preterm labor process. Our study has demonstrated that the sampling is easy to perform and is highly reliable and reproducible. The combination of effective screening with an efficient intervention may open new perspectives for prevention of pregnancy complications, including preterm birth.

## Figures and Tables

**Table 1:** Number (*N*, %) of positive samples per location and per trimester in qPCR+ patients.

Sampling location	qPCR + T1 (*N* = 55)	qPCR + T2 (*N* = 32)	qPCR + T3 (*N* = 29)	Total number of tests (*N* = 116)
Vagina (vg)	55 (100%)	32 (100%)	28 (96.6%)	115 (99.1%)
Fornix (fx)	52 (97.5%)	30 (93.8%)	27 (93.1%)	109 (94.0%)
Cervical 1 (cx1)	47 (85.5%)	30 (88.2%)	27 (93.1%)	104 (89.7%)
Cervical 2 (cx2)	44 (80.0%)	27 (84.4%)	26 (87.7%)	97 (83.6%)

**Table 2:** Qualitative reproducibility during first trimester.

	*κ* coefficient
vg/fx	0.95
vg/cx1	0.87
vg/cx2	0.84
fx/cx1	0.87
fx/cx2	0.83
cx1/cx2	0.93

vg: vaginal; fx: fornix; cx: cervical.

## Additional Points

*Condensation*. Effect of genital sampling site on* Ureaplasma *spp. qPCR results.
